# Gangrenous Intestine—Resection—Anastomosis with the Murphy Button—Recovery

**Published:** 1898-02

**Authors:** Frank A. Jones

**Affiliations:** Rochester, N. Y.


### GANGRENOUS INTESTINE—RESECTION—ANASTOMOSIS WITH THE MURPHY BUTTON—RECOVERY.

BY FRANK A. JONES, M. D., Rochester, N. Y.

I WAS called July 16, 1897, to see Mrs. S., a widow 27 years of age, mother of one child, of previous good health. She was suffering from very severe pain in the right iliac region, with very little, if any, distension noticeable, very slight tenderness on pressure, pulse and temperature normal. Bimanual examination disclosed a mass about the size of an orange, which I concluded was an enlarged ovary. Bowels were regular and menses normal. Large hypodermics of morphia were required, and at times it became necessary to administer chloroform. The following morning she was so much improved that she walked to the bath-room. Pains increased by night, however, and her pulse became accelerated, temperature now 101°, nausea and vomiting present, abdomen distended. I had her removed to the hospital, where, on the 18th of July, after due preparation, she was put under chloroform, and on opening the abdomen I found a cystic tumor of the right broad ligament which had ruptured at the pedicle, filling the abdomen with blood and clots ; this was ligated and removed, abdomen was thoroughly cleansed and closed. She made a good recovery and returned to her home in four weeks, and in a short time was well and strong, assisting with the house-work. And after a long walk on October 2d was suddenly seized on the morning of October 3d, at five o'clock, with pain of the same severity on the left side, on a line with the umbilicus. Nothing could be made out by vaginal or rectal examination, bowels had been moving freely up to the day previous, since which time there had been no evacuation nor gas. Large enemas were given with no result. It was simply impossible to keep her comfortable without chloroform. Pulse and temperature were normal, no nausea, no vomiting, no regurgitations, very little distension or tenderness on pressure. This condition continued until the following morning, when I was called, and found her apparently in a collapse, no pulse at the wrist, respirations very rapid, skin cold and clammy, absence of pain, eyes sunken, face pinched, and, all in all, a complete picture of despair. Abdomen was now distended and tympanitic. Strychnia, nitro-glycerine and brandy were used hypodermically, also infusions of salt solution under the skin in immense quantities. She began to rally about noon and at four o'clock, everything having been prepared for an operation, she was placed upon the table, pulse at that time very feeble and about 180. A small amount of chloroform was administered when I cut down through the old incision and found a large mass of gangrenous small intestine. As rapidly as possible this mass was removed and anastomosis made with a medium-sized Murphy button. The abdominal toilet was completed as soon as possible, a drainage-tube introduced and abdomen was closed with silkworm-gut sutures and patient placed in bed. She rallied at once after being placed in bed, suffered from no shock, no vomiting, pulse dropped to 128 in less than twenty-four hours, bowels moved, a large amount of gas was expelled, and they continued to move freely from one to four times each day. It was found by actual measurement that thirty-two inches of small intestine had been removed. Pulse fell to 80, temperature normal, which had never exceeded $99\frac{4}{5}°$. She continued to improve from day to day and the button was recovered on the fifteenth day. She then made an uninterrupted recovery, and is now, after two months, able to attend to her household duties.

155 Lake Avenue.

